# Low Free Triiodothyronine as a Predictor of Poor Prognosis in Patients With Myocardial Infarction With Non-Obstructive Coronary Arteries

**DOI:** 10.3389/fendo.2021.681978

**Published:** 2021-05-31

**Authors:** Fuad A. Abdu, Abdul-Quddus Mohammed, Lu Liu, Wen Zhang, Guoqing Yin, Bin Xu, Siling Xu, Yawei Xu, Wenliang Che

**Affiliations:** ^1^Department of Cardiology, Shanghai Tenth People’s Hospital, Tongji University School of Medicine, Shanghai, China; ^2^Department of Cardiology, Shanghai Tenth People’s Hospital Chongming Branch, Shanghai, China

**Keywords:** MINOCA, thyroid, low free triiodothyronine level, prognosis, predictors

## Abstract

**Background:**

Low free triiodothyronine (fT3) level is strongly associated with poor prognosis in various patient populations. However, the role of fT3 in the risk of clinical outcomes in myocardial infarction with non-obstructive coronary arteries (MINOCA) has not been studied. Our study aimed to evaluate the association between low fT3 levels and the clinical outcomes of MINOCA patients.

**Methods:**

A total of 218 MINOCA patients without a history of thyroid disease were enrolled in the study. Demographic, baseline clinical data, thyroid hormones, and other biochemical parameters were assessed in all patients. According to the fT3 levels, the present study was classified into two groups: the low fT3 group (fT3<3.5 pmol/L) and the normal fT3 group (fT3 3.5-6.5 pmol/L). The endpoint of the study was major adverse cardiac events (MACE).

**Results:**

Fifty-nine patients were in the low fT3 group and 159 patients were in the normal fT3 group. Over the two years of follow-up, 36 MACE have occurred. The occurrence of MACE was higher in the low fT3 group compared with normal fT3 group (25.4% vs 13.2%; P=0.031). Kaplan-Meier survival curves showed a significantly increased risk of MACE in patients with low fT3 (log-rank P=0.027). Multivariable logistic regression analysis stated that high fT3 was independently associated with lower risk of MACE after two years of follow up (OR, 0.623; 95% CI, 0.399- 0.972; P=0.037).

**Conclusion:**

Low fT3 levels were significantly associated with increased risk of MACE in patients with MINOCA. This finding suggests that the fT3 levels may serve as a potential biomarker in risk stratification of MINOCA patients.

## Introduction

With the continuous improvement in understanding of acute myocardial infarction (AMI), a group of AMI patients with no angiographic obstructive CAD (stenosis<50%) were gradually discovered, and the term myocardial infarction with non-obstructive coronary arteries (MINOCA) was given for this illness (1, 2). MINOCA represents a group of heterogeneous disorder with various pathological mechanisms (3, 4), and it is not a benign disease, the one-year mortality rate is 4.7% (5), however, the five-year mortality exceeds 10.9% (6), as well as a major adverse cardiac event (MACE) occurs in one in five patients with MINOCA over one year (7). Considering the heterogeneity and poor prognosis of MINOCA patients, it might be necessary to accurately use clinical predictors to stratify patients with varying risks of new cardiovascular events to help clinicians to develop adequate management strategies and to reduce the occurrence of MACE in such patient population.

There is a strong relation between thyroid hormones (THs) especially the active hormone triiodothyronine (T3) and cardiovascular system (8, 9). T3 plays a significant role in modulating cardiac contractility, increasing heart rate, and decreasing arterial resistance (9, 10). Low levels of free triiodothyronine (fT3) are correlated with worse prognosis in various patient populations such as cardiac patients (10), heart failure (11–13), AMI (14, 15), acute myocarditis (16), coronary artery bypass grafting (CABG) (17), acute ischaemic stroke (18), and in other groups of patients (19–24), however, the role of fT3 in the risk of clinical outcomes in MINOCA population and whether fT3 can improve risk prediction of MACE in such patients group has not been evaluated.

Therefore, the purpose of this study was to evaluate the association between low fT3 levels and the clinical outcomes of MINOCA patients.

## Methods

### Study Population and Participants

This was an observational study of 218 consecutive patients from 2014 to 2018 diagnosed with MINOCA and undergoing coronary angiography (CAG) at the department of cardiology, Shanghai Tenth People’s Hospital.

The inclusion criterion of the present study was (1): A diagnosis of MINOCA according to the ESC guidelines (2) and the fourth universal definition of myocardial infarction guidelines (1), which include: complies with the AMI’s diagnostic criteria (1); CAG showed non-obstructive coronary disease (< 50% stenosis) in any infarct related coronary artery; there is no other obvious clinically evident explanation which may explain the acute presentation such as myocarditis or pulmonary embolism (2); Age >18 years old.

The exclusion criteria were (1) Patients with thrombolytic therapy given before CAG, patients with a history of MI or coronary intervention (2), Patients with severe liver, kidney disorders and had a malignant tumor (3), Patients with thyroid disorder history, such as hyperthyroidism, hypothyroidism, or thyroiditis (4), Patients who are receiving thyroid-related medications, or (5) Patients without THs baseline data.

Our study complied with the Helsinki Declaration and was approved by the hospital’s ethical review board (Shanghai Tenth People’s Hospital, Tongji University, Shanghai, China). Informed written consent was received from all patients participating in this study.

### Data Collection

Demographic and baseline clinical information (such as age, sex, smoking history, hypertension, diabetes mellitus, body mass index, hyperlipidemia, heart failure, atrial fibrillation, heart rate, and blood pressure) were meticulously documented after admission. The electrocardiogram (ECG) and echocardiography (Echo) were performed in all the patients. All participants were undergoing the CAG procedure after admission.

### Biochemical Assessment

Fasting blood was obtained within 24 hours of admission to analyze biochemical parameters. The serum levels of THs [including fT3, total triiodothyronine (TT3), free tetraiodothyronine (fT4), total tetraiodothyronine (TT4), and thyroid stimulating hormone (TSH)], and serum cardiac biomarkers [including troponin-T (cTnT), myoglobin, creatine kinase-MB (CK-MB), and N-terminal pro-brain natriuretic peptide (NT-proBNP)] were measured in all patients. As described previously (25), the serum levels of THs in our department were measured using chemiluminescence (Automatic Chemiluminescence Immune Assay System ACS 180 with related kits; Bayer, Berlin, Germany). The levels of NT-ProBNP were measured using the Eleusis electro-chemiluminescent immunoassay (Roche Diagnostics Ltd. Rotkreuz, Switzerland).

The normal ranges for fT3, TT3, fT4, TT4, and TSH uses in our department are 3.5-6.5 pmol/L, 1.2-3.4 nmol/L,10.2-31 pmol/L, 4-174 nmol/L, and 0.35-5.5 mIU/L, respectively (25).

According to fT3 level at admission, the present study was classified into two groups: low fT3 group (fT3 < 3.5 pmol/L) and normal fT3 group (fT3 3.5-6.5 pmol/L).

### Follow-Up and Endpoints

Follow-up started from the day of admission and was performed at 3, 6, 12 months, and 2 years after discharge by interviewing the patients by trained cardiologists at the Shanghai Tenth People’s Hospital. Follow-up information was carried out through outpatient visits, telephone calls, reviewing electronic medical records, and clinical notes.

The primary endpoint of our study was MACE, described as cardiovascular death, heart failure, nonfatal MI, stroke, and angina rehospitalization. The definitions of concepts in the primary endpoint have been described in our previous study (26). Death from ACS, severe cardiac arrhythmia, or refractory congestive heart failure was defined as cardiovascular death. The diagnosis of nonfatal MI was based on the established guidelines for myocardial infarction (1). Stroke was defined as an ischemic cerebral infarction caused by embolic or thrombotic occlusion of a major intracranial artery. Heart failure is a progressive disorder identified by severe forms, which might be followed by symptoms of structural and/or functional cardiac abnormality, culminating in decreased cardiac output and/or increased intracardiac pressure during rest or stress.

### Statistical Analysis

The data in this study were analyzed using the Statistical Package for Social Sciences (SPSS) v.22. Figures were formed by using GraphPad softwarev.8.0.1. For numerical variables, the mean ± SE with a normal distribution was used, and percentages (%) were used for categorical variables. The chi-square test and Fisher’s exact tests were used to compare categorical variables. An independent sample t-test was used to compare numerical variables between groups. Logistic regression analysis was used to determine the adjusted odds ratio (OR) for MACE to evaluate predictors of clinical endpoints. Sex, age, traditional cardiovascular risk factors (BMI, smoking history, diabetes, hypertension, hyperlipidaemia, atrial fibrillation, heart failure and alcohol), systolic and diastolic blood pressure, heart rate, left ventricular ejection fraction (LVEF), ECG findings, angiographic characteristics, and biochemical parameters (cTnT, CK-MB, myoglobin, NT-proBNP, and THs) were considered as covariates in the univariate models. For the multivariable models, clinical risk factors and biochemical parameters which were univariate predictors (at P < 0.10) were considered as covariates. Further subgroup analysis was performed to determine the interactions between fT3 levels and clinically associated variables using Cox proportional hazards analysis. The Kaplan-Meier analysis was used to evaluate MACE-free survival rates, and the differences between the two groups were determined using the log-rank test. The ability of fT3 to predict MACE in MINOCA patients was displayed using receiver operating characteristic (ROC) curves analysis. All analysis was conducted two-sided and identified statistically significant at P-value < 0.05.

## Results

### Baseline Characteristics

A total of 218 patients who met the diagnostic criteria of MINOCA were enrolled in the present study. Among these, 59 (27.1%) were in the low fT3 group and 159 (72.9%) were in the normal fT3 group. Baseline characteristics, laboratory findings, and angiographic data of low and normal fT3 are summarized in **Tables 1**, **2**. Patients with low fT3 levels were older (70.18 ± 12.96 vs. 60.77 ± 13.01, p < 0.001), whereas systolic and diastolic blood pressure in normal fT3 was higher (P < 0.05). Echocardiography data showed that the LVEF in the normal fT3 group was higher than the low fT3 group (56.49% vs. 48.75%, P < 0.001). The serum levels of fT3, TT3, fT4, and TT4 in the low fT3 group were lower (P < 0.05); by comparison, the levels of cardiac cTnT, CK-MB, myoglobin, and NT-proBNP in the low fT3 group were significantly higher than normal fT3 group. According to CAG results, patients with 3 vessels disease were more common in the low fT3 group (16.9% vs. 3.8%, P = 0.002). There were no significant differences in sex, smoking history, diabetes, hypertension, hyperlipidemia, atrial fibrillation, previous heart failure, alcohol use, and body mass index between the two groups (P > 0.05).

**Table 1:** Baseline characteristics of the study population.

	Low fT3 (*n* = 59)	Normal fT3 (*n* = 159)	*P* value
Age (years)	70.18 ± 12.96	60.77 ± 13.01	<0.001
Female, n (%)	34 (57.6)	74 (46.5)	0.146
BMI (kg/m2)	23.36 ± 4.59	24.37 ± 3.63	0.111
Smoking history, n (%)	21 (35.6)	62 (39.0)	0.646
Diabetes, n (%)	8 (13.6)	25 (15.7)	0.692
Hypertension, n (%)	32 (54.2)	75 (47.2)	0.354
Hyperlipidaemia, n (%)	7 (11.9)	31 (19.5)	0.187
Atrial fibrillation, n (%)	8 (13.6)	13 (8.2)	0.231
Previous heart failure, n (%)	2 (3.4)	4 (2.5)	0.726
Alcohol use	5 (8.5)	18 (11.3)	0.543
LVEF (%)	48.75 ± 13.69	56.49 ± 11.01	<0.001
STEMI, n (%)	25 (42.4)	57 (35.8)	0.377
NSTEMI, n (%)	34 (57.6)	102 (64.2)	0.377
Systolic blood pressure (mmHg)	135.31 ± 25.49	142.98 ± 23.25	0.036
Diastolic blood pressure (mmHg)	75.06 ± 13.22	82.13 ± 13.10	0.001
Heart rate, beats per minute	84.81 ± 23.38	80.22 ± 17.58	0.121

Values are expressed as mean ± SD or number (%); BMI, body mass index; LVEF, left ventricular ejection fraction; STEMI, ST-segment elevation myocardial infarction; NSTEMI, non-ST-elevation myocardial infarction; fT3, free triiodothyronine.

**Table 2:** Laboratory findings and angiographic data of the study population.

	Low fT3 (*n* = 59)	Normal fT3 (*n* = 159)	*P* value
**laboratory findings**			
fT3 (pmol/L)	2.95 ± 0.49	4.59 ± 1.67	<0.001
TT3 (nmol/L)	0.99 ± 0.28	1.52 ± 0.46	<0.001
fT4 (pmol/L)	14.58 ± 3.33	16.59 ± 5.08	0.005
TT4 (nmol/L)	86.79 ± 24.28	100.34 ± 30.14	0.002
TSH (mIU/L)	4.30 ± 19.36	1.97 ± 1.50	0.133
cTnT (ng/mL)	0.69 ± 1.64	0.38 ± 0.77	0.053
CK-MB (ng/mL)	30.82 ± 75.98	14.43 ± 27.39	0.020
Myoglobin (ng/ml)	285.63 ± 471.78	99.15 ± 164.94	<0.001
NT-proBNP (pg/mL)	4731.77 ± 6846.12	1209.66 ± 2681.39	<0.001
**Angiographic data**			
Normal coronary arteries (0% stenosis), n (%)	22 (37.3)	76 (47.8)	0.166
Vessel with any stenosis (> 0 to < 50% stenosis), n (%)			
1-vessel, n (%)	19 (32.2)	46 (28.9)	0.639
2-vessel, n (%)	8 (13.6)	31 (19.5)	0.310
3-vessel, n (%)	10 (16.9)	6 (3.8)	0.002

Values are expressed as mean ± SD or number (%); fT3, free triiodothyronine; TT3, total triiodothyronine; fT4, free tetraiodothyronine; TT4, total tetraiodothyronine; TSH, thyroid stimulating hormone; cTnT, cardiac troponin T; CK-MB, creatine kinase isoenzyme; NT-proBNP, N-terminal pro-brain natriuretic peptide.

### Association Between fT3 and the Primary Endpoints

**Figure 1:**
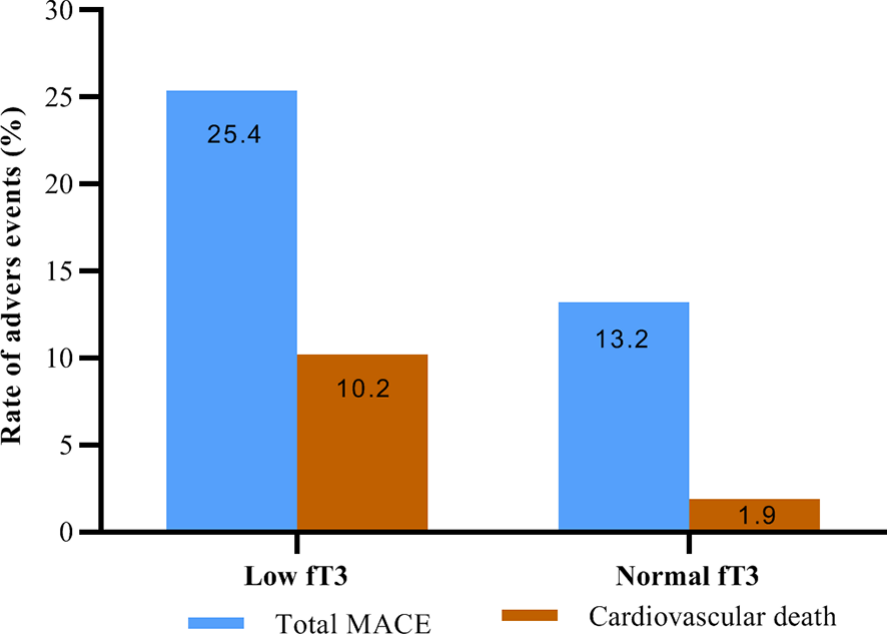
Rate of adverse events in MINOCA patients with low fT3 versus normal fT3 levels. fT3, free triiodothyronine; MACE, major adverse cardiovascular events.

**Figure 2:**
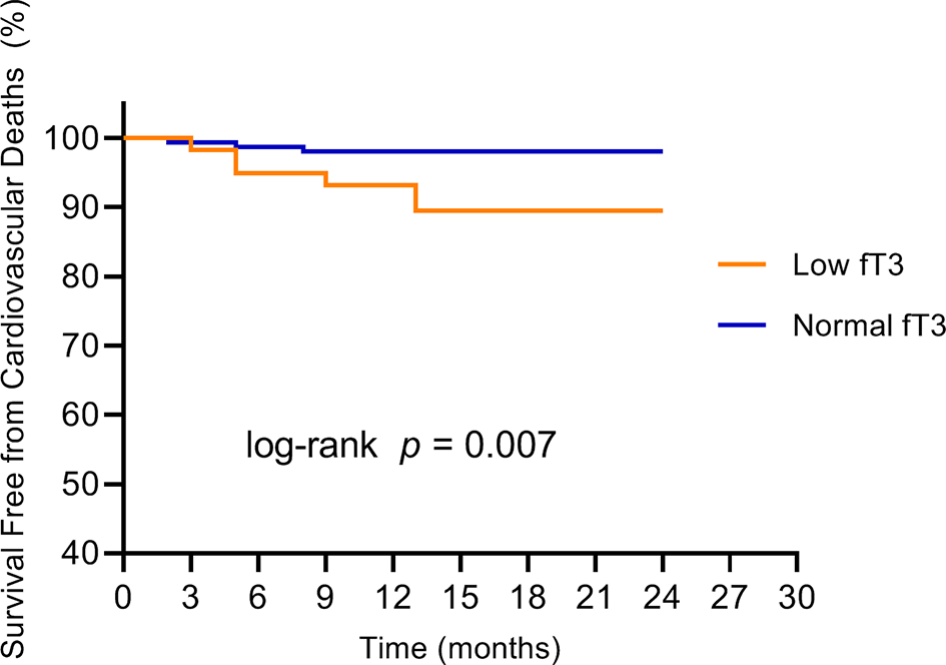
Kaplan-Meier survival curves for cardiovascular deaths in MINOCA patients with low fT3 versus normal fT3 levels. fT3, free triiodothyronine.

**Figure 3:**
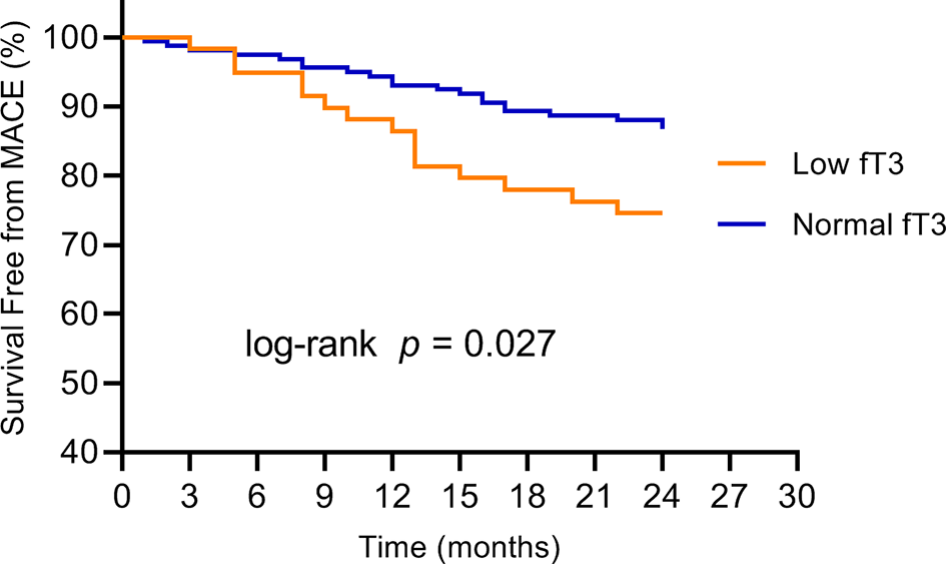
Kaplan-Meier survival curves for MACE in MINOCA patients with low fT3 versus normal fT3 levels. fT3, free triiodothyronine; MACE, major adverse cardiovascular events.

**Figure 4:**
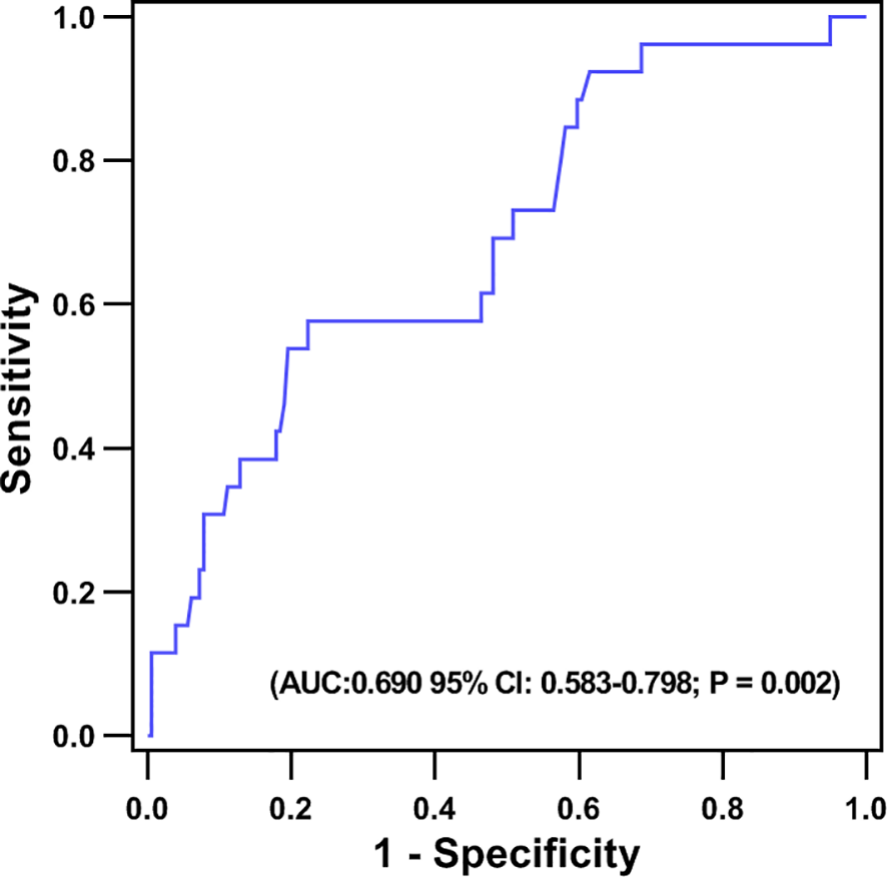
Receiver operating characteristic (ROC) curve of the ability of fT3 to predict MACE in MINOCA patients.

No patients were excluded because of missing data. Over the two years of follow-up, a total of 36 MACE have occurred. MACE occurred in 15 patients (6 cardiovascular deaths, 1 heart failure, 1 stroke, and 7 angina rehospitalization) in the low fT3 group, and 21 patients (3 cardiovascular deaths, 2 nonfatal MI, 1 heart failure, 2 strokes, and 13 angina rehospitalization) in the normal fT3 group. The occurrence of MACE and cardiovascular deaths were substantially more frequent in the low fT3 group compared with the normal fT3 group (25.4% vs 13.2% and 10.2% vs. 1.9%, respectively; all P < 0.05) (**Figure 1**). Two years Kaplan-Meier survival curves for cardiovascular deaths and total MACE in patients with low and normal fT3 levels are displayed in **Figures 2**, **3**, which showed a significantly increased risk of cardiovascular deaths in patients with low fT3 (log-rank P = 0.007). Similarly, when total MACE was analyzed, the Kaplan-Meier analysis demonstrated a higher risk of total MACE in low fT3 (log-rank P = 0.027). The ROC curve of fT3 was shown in **Figure 4** for the prediction of clinical MACE, which showed that the fT3 has moderate significance in predicting MACE in MINOCA patients, with an AUC of 0.690 (95% CI, 0.583-0.798; P = 0.002).

### Predictive Factors of MACE

The univariable and multivariable predictors of MACE are included in **Table 3**. Logistic regression analysis demonstrates that high fT3 in univariate analysis was independently associated with lower risk of MACE (OR, 0.647; 95% CI, 0.422- 0.991; P = 0.045). After excluding confounding factors, multivariable logistic regression analysis still stated that high fT3 was strongly associated with lower risk of MACE after two years of follow up (OR, 0.623; 95% CI, 0.399- 0.972; P = 0.037), followed by LVEF (OR, 0.957; 95% CI, 0.928- 0.986; P = 0.004).

**Table 3:** Univariate and Multivariable analysis of predictors of MACE within 2 years.

Variable	Univariate analysis		Multivariable analysis	
	*OR* (95% CI)	*P* value	*OR* (95% CI)	*P* value
fT3	0.647 (0.422- 0.991)	0.045	0.623 (0.399- 0.972)	0.037
NT-proBNP	1.000 (1.000- 1.000)	0.053		
TT3	0.398 (0.148- 1.070)	0.068		
LVEF	0.950 (0.926- 0.976)	<0.001	0.957 (0.928- 0.986)	0.004
Age	1.031 (1.002- 1.061)	0.036		
Systolic blood pressure	1.017 (1.001- 1.032)	0.032		

fT3, free triiodothyronine; NT-proBNP, N-terminal pro-brain natriuretic peptide; TT3, total triiodothyronine; LVEF, left ventricular ejection fraction; CI, confidence interval; OR, odds ratio.

### Subgroup Analysis

Further subgroup analyses for associations of fT3 levels with MACE stratified by age, sex, traditional cardiovascular risk factors, LVEF, cTnT, NT-proBNP levels, and the number of vessel diseases are shown in supplemental **Table 1**. Subgroup analysis showed that there was no significant interaction between fT3 levels and age (<65 years or ≥65 years), sex, traditional cardiovascular risk factors (hypertension, diabetes mellitus, smoking, BMI, and atrial fibrillation), LVEF, cTnT, NT-proBNP levels, and the number of vessel diseases (all P for interaction > 0.05).

## Discussions

The aim of this study was to evaluate the association between low fT3 levels and the clinical outcomes of MINOCA patients. The main findings of our study were (1); Low fT3 levels were frequently found in MINOCA patients (2), Low fT3 levels were strongly associated with increased risk of MACE in MINOCA patients. The findings of this study suggest that measuring fT3 levels may be a useful tool for clinicians to stratify patients at risk of poor outcomes following MINOCA.

The importance of MINOCA has attracted much attention and has been recently introduced in the European Society of Cardiology (ESC) (2) and the Fourth Universal Definition of Myocardial Infarction guidelines (1) as a special type of myocardial infarction (MI). Early large cohort studies indicated that the prognosis of MINOCA patients is not favorable. A large cohort study of 16849 MINOCA patients reported that one in every five MINOCA patients experienced a major adverse event over one year (7). Furthermore, Lindahl et al. concluded that 23.9% of MINOCA patients suffered MACE over a four-year follow-up period in a study of 9466 MINOCA patients (27). Consequently, MINOCA remains a particularly challenging illness due to numerous pathophysiological mechanisms with different causes and unclear management therapy. Thus, clinical predictors can be used to guide the medical triage process and to quantify the risk of major cardiovascular events in the MINOCA population, which may be beneficial to improve treatment options.

Low T3 levels are often seen in severe non-thyroidal diseases, such as cardiovascular disorders (10). According to a recent systemic review, the incidence of low T3 syndrome was 18.9%, 24.5%, and 17.1% in patients with MI, heart failure, and acute coronary syndrome (ACS), respectively (28). In the present study, the prevalence of low fT3 in MINOCA patients was 27.1%, which is relatively high. Interestingly, the level of myocardial injury markers (cTnT, CK-MB, and myoglobin) and NT-proBNP were higher in the low fT3 group. Moreover, LVEF in the low fT3 group was lower, besides, patients with the three-vessel disease were more common in the low fT3 group, suggesting that fT3 level is associated with the degree of myocardial injury and severity in MINOCA patients. Similar findings were observed in a study by Yuan et al, which indicates that a low level of fT3 was linked with an increased incidence of long-term cardiovascular mortality and composite MACE in euthyroid patients with the three-vessel disease (29). Thus, low fT3 could possibly relate to sicker patients and reflect non-thyroidal illness.

Previous studies have demonstrated that low fT3 levels were associated with a worse short and long-term prognosis in various patient groups. A study by Iervasi et al. showed that low fT3 is a strong predictor of mortality in 573 patients with different cardiac diseases (10). Accordingly, another study of 699 STEMI patients showed low fT3 levels were significantly associated with increased risks of mortality and MACE (14). Low fT3 was also found to be a significant predictor of poor prognosis in a recent Chinese study in adult patients with acute myocarditis (16). In 2459 AMI patients, Wen Su et al. also found a strong link between poor prognosis and low fT3 syndrome (15). Another study observed that low T3 is a powerful factor of mortality and low cardiac output in CABG patients (17). A recent Japanese study demonstrated low fT3 to be a predictor of MACE and all-cause mortality in hemodialysis patients (30). Multiple studies have reported low T3 levels to be a predictor of poor prognosis in acute and chronic heart failure (12, 13, 31, 32). In patients with acute ischaemic stroke, low T3 syndrome was associated with hemorrhagic transformation (18). Low T3 was found to be correlated with poor prognosis in some cancer diseases such as chronic lymphocytic leukemia (20), brain tumor (33), and diffuse large B cell lymphoma (21). Besides, Low T3 was also reported to be a useful predictor of poor clinical outcomes in patients with pyogenic liver abscess (19), chronic fatigue syndrome (22), community-acquired pneumonia (23), secondary hemophagocytic lymph histiocytosis (24), and respiratory failure (34). In a community dwelling elderly population, low fT3 was linked to low muscle mass and poor physical ability (35). In MINOCA patients, two recent case studies have shown that thyroid diseases such as thyrotoxicosis may be a potential cause for MINOCA, suggesting that thyroid hormones may play an important role in the pathogenesis of MINOCA and may have an impact on the prognosis of such patients group (36, 37). To the best of our knowledge, this is the first study to demonstrate a relationship between lower fT3 levels and increased risk of MACE and cardiovascular deaths in the MINOCA population. In the present study, the occurrence of MACE and cardiovascular deaths were more frequent in the low fT3 group compared to the normal fT3 group. The Kaplan-Meier analysis also showed a significantly increased risk of cardiovascular deaths and total MACE in MINOCA patients with low fT3. Furthermore, multivariable logistic regression analysis stated that fT3 was significantly associated with poor prognosis in the MINOCA population, this correlation was consistent throughout subgroups of the patient’s population. In addition, the ROC curve analysis showed that the fT3 has moderate significance in predicting MACE in MINOCA patients. Considering the poor prognosis of MINOCA patients, it might be necessary to use clinical predictors to stratify patients with varying risks of new cardiovascular events to help clinicians develop adequate management strategy and to reduce the occurrence of MACE. Thus, the findings of the present study are of significant clinical interest, which suggests that determination of fT3 levels in the early stage could be a useful tool to identify the risk of poor clinical outcomes and improve the management strategy of high-risk MINOCA patients. The assessment of fT3 levels in cardiovascular risk scores of MINOCA patients may provide significant information on clinical implications, however, these findings require further verification by large scale prospective cohorts.

Some limitations were associated with our research study. First, this was an observational study with small sample size. Second, this study has only two years follow-up duration. Third, some confounders such as a family history of heart disease, etc., might have influenced our findings in few patients, therefore, the results of the present study may not be generalizable to populations of different ethnic backgrounds. Fourth, we do not have data on autoantibodies, and the frequency of autoimmune thyroid conditions in this study is unclear. In addition, since thyroid function was only assessed at baseline, we cannot conclude that the associations with MACE would be different if data were collected over the follow-up period. Furthermore, it is unclear if increasing the fT3 level into its normal range would benefit patients with low fT3 to improve their outcomes. Multi-center prospective analyses with long-term follow-up are needed to verify the findings of the present study.

## Conclusion

The present study is the first to evaluate the association between low fT3 levels and the prognosis of MINOCA patients. Our study revealed that low fT3 levels are frequently found in MINOCA patients and were significantly associated with increased risk of MACE. This finding suggests that the fT3 levels may serve as a potential biomarker in the risk stratification of MINOCA patients.

## Data Availability Statement

The raw data supporting the conclusions of this article will be made available by the authors, without undue reservation.

## Ethics Statement

The studies involving human participants were reviewed and approved by the hospital’s ethical review board (Shanghai Tenth People’s Hospital, Tongji University, Shanghai, China). The patients/participants provided their written informed consent to participate in this study.

## Author Contributions

FA, YX, and WC designed the study. A-QM, LL, WZ, and GY collected the data. BX and SX. were involved in data cleaning, follow-up, and verification. FA, YX, and WC drafted the manuscript and revised it critically for important intellectual content. YX and WC approved the final version of the manuscript. All authors contributed to the article and approved the submitted version.

## Funding

This work was supported by the Fundamental Research Funds for Central Universities (NO.22120190211), Foundation of Chongming (CKY2018-18, CKY2020-29), and Clinical Research Plan of SHDC (SHDC2020CR4065).

## Conflict of Interest

The authors declare that the research was conducted in the absence of any commercial or financial relationships that could be construed as a potential conflict of interest.

## Abbreviations

AMI, acute myocardial infarction; MINOCA, myocardial infarction with non-obstructive coronary arteries; MACE, major adverse cardiac events; THs, thyroid hormones; T3, hormone triiodothyronine; fT3, free triiodothyronine; CAG, coronary angiography; TT3, total triiodothyronine; fT4, free tetraiodothyronine; TT4, total tetraiodothyronine; TSH, thyroid stimulating hormone; cTnT, cardiac troponin T; CK-MB, creatine kinase isoenzyme; NT-proBNP, N-terminal pro-brain natriuretic peptide; BMI, body mass index; LVEF, left ventricular ejection fraction; CI, confidence interval; OR, odds ratio; ROC, receiver operating characteristic.

## References

[1] Thygesen K Alpert J Jaffe A Chaitman B Bax J Morrow D. Fourth Universal Definition of Myocardial Infarction (2018). J Am Coll Cardiol (2018) 72(18):2231–64. 10.1016/j.jacc.2018.08.1038 30153967

[2] Agewall S Beltrame JF Reynolds HR Niessner A Rosano G Caforio ALP. ESC Working Group Position Paper on Myocardial Infarction With non-Obstructive Coronary Arteries. Eur Heart J (2016) 38(3):143–53. 10.1093/eurheartj/ehw149 28158518

[3] Tamis-Holland J Jneid H Reynolds H Agewall S Brilakis E Brown T. Contemporary Diagnosis and Management of Patients With Myocardial Infarction in the Absence of Obstructive Coronary Artery Disease: A Scientific Statement From the American Heart Association. Circulation (2019) 139(18):e891–908. 10.1161/cir.0000000000000670 30913893

[4] Niccoli G Scalone G Crea F. Acute Myocardial Infarction With No Obstructive Coronary Atherosclerosis: Mechanisms and Management. Eur Heart J (2015) 36(8):475–81. 10.1093/eurheartj/ehu469 25526726

[5] Pasupathy S Air T Dreyer R Tavella R Beltrame J. Systematic Review of Patients Presenting With Suspected Myocardial Infarction and Nonobstructive Coronary Arteries. Circulation (2015) 131(10):861–70. 10.1161/circulationaha.114.011201 25587100

[6] Bainey KR Welsh RC Alemayehu W Westerhout CM Traboulsi D Anderson T. Population-Level Incidence and Outcomes of Myocardial Infarction With non-Obstructive Coronary Arteries (MINOCA): Insights From the Alberta Contemporary Acute Coronary Syndrome Patients Invasive Treatment Strategies (COAPT) Study. Int J Cardiol (2018) 264:12–7. 10.1016/j.ijcard.2018.04.004 29655952

[7] Dreyer R Tavella R Curtis J Wang Y Pauspathy S Messenger J. Myocardial Infarction With Non-Obstructive Coronary Arteries as Compared With Myocardial Infarction and Obstructive Coronary Disease: Outcomes in a Medicare Population. Eur Heart J (2020) 41(7):870–8. 10.1093/eurheartj/ehz403 PMC7778433 31222249

[8] Klein I Ojamaa K. Thyroid Hormone and the Cardiovascular System. N Engl J Med (2001) 344(7):501–9. 10.1056/nejm200102153440707 11172193

[9] Jabbar A Pingitore A Pearce SH Zaman A Iervasi G Razvi S. Thyroid Hormones and Cardiovascular Disease. Nat Rev Cardiol (2017) 14(1):39–55. 10.1038/nrcardio.2016.174 27811932

[10] Iervasi G Pingitore A Landi P Raciti M Ripoli A Scarlattini M. Low-T3 Syndrome: A Strong Prognostic Predictor of Death in Patients With Heart Disease. Circulation (2003) 107(5):708–13. 10.1161/01.cir.0000048124.64204.3f 12578873

[11] Passino C Pingitore A Landi P Fontana M Zyw L Clerico A. Prognostic Value of Combined Measurement of Brain Natriuretic Peptide and Triiodothyronine in Heart Failure. J Card Fail (2009) 15(1):35–40. 10.1016/j.cardfail.2008.08.008 19181292

[12] Rothberger GD Gadhvi S Michelakis N Kumar A Calixte R Shapiro LE. Usefulness of Serum Triiodothyronine (T3) to Predict Outcomes in Patients Hospitalized With Acute Heart Failure. Am J Cardiol (2017) 119(4):599–603. 10.1016/j.amjcard.2016.10.045 28017303

[13] Sato Y Yoshihisa A Kimishima Y Kiko T Kanno Y Yokokawa T. Low T3 Syndrome Is Associated With High Mortality in Hospitalized Patients With Heart Failure. J Card Fail (2019) 25(3):195–203. 10.1016/j.cardfail.2019.01.007 30682427

[14] Song Y Li J Bian S Qin Z Song Y Jin J. Association Between Low Free Triiodothyronine Levels and Poor Prognosis in Patients With Acute St-Elevation Myocardial Infarction. BioMed Res Int (2018) 2018:9803851. 10.1155/2018/9803851 29850596 PMC5926512

[15] Su W Zhao XQ Wang M Chen H Li HW. Low T3 Syndrome Improves Risk Prediction of In-Hospital Cardiovascular Death in Patients With Acute Myocardial Infarction. J Cardiol (2018) 72(3):215–9. 10.1016/j.jjcc.2018.02.013 29580665

[16] Zhao Y Wang W Zhang K Tang Y-D. Association Between Low T3 Syndrome and Poor Prognosis in Adult Patients With Acute Myocarditis. Front Endocrinol (2021) 12:571765. 10.3389/fendo.2021.571765 PMC7984427 33763025

[17] Cerillo AG Storti S Kallushi E Haxhiademi D Miceli A Murzi M. The Low Triiodothyronine Syndrome: A Strong Predictor of Low Cardiac Output and Death in Patients Undergoing Coronary Artery Bypass Grafting. Ann Thorac Surg (2014) 97(6):2089–95. 10.1016/j.athoracsur.2014.01.049 24636708

[18] Huang GQ Zeng YY Cheng QQ Cheng HR Ruan YT Yuan CX. Low Triiodothyronine Syndrome Is Associated With Hemorrhagic Transformation in Patients With Acute Ischaemic Stroke. Aging (2019) 11(16):6385–97. 10.18632/aging.102195 PMC6738409 31454331

[19] Xu J Wang L. Low T3 Syndrome as a Predictor of Poor Prognosis in Patients With Pyogenic Liver Abscess. Front Endocrinol (2019) 10:541. 10.3389/fendo.2019.00541 PMC6691090 31447784

[20] Gao R Chen RZ Xia Y Liang JH Wang L Zhu HY. Low T3 Syndrome as a Predictor of Poor Prognosis in Chronic Lymphocytic Leukemia. Int J Cancer (2018) 143(3):466–77. 10.1002/ijc.31327 29457831

[21] Gao R Liang JH Wang L Zhu HY Wu W Wu JZ. Low T3 Syndrome Is a Strong Prognostic Predictor in Diffuse Large B Cell Lymphoma. Br J Haematol (2017) 177(1):95–105. 10.1111/bjh.14528 28146267

[22] Ruiz-Núñez B Tarasse R Vogelaar EF Janneke Dijck-Brouwer DA Muskiet FAJ. Higher Prevalence of “Low T3 Syndrome” in Patients With Chronic Fatigue Syndrome: A Case-Control Study. Front Endocrinol (2018) 9:97. 10.3389/fendo.2018.00097 PMC5869352 29615976

[23] Liu J Wu X Lu F Zhao L Shi L Xu F. Low T3 Syndrome Is a Strong Predictor of Poor Outcomes in Patients With Community-Acquired Pneumonia. Sci Rep (2016) 6:22271. 10.1038/srep22271 26928863 PMC4772089

[24] Yin G Huang J Tian T Duan L Xu J Qiu H. Low T3 Syndrome Is a Prognostic Marker of Poor Outcomes in Secondary Hemophagocytic Lymphohistiocytosis. Leuk Lymphoma (2020) 61(12):2947–54. 10.1080/10428194.2020.1789623 32643969

[25] Zhang B Peng W Wang C Li W Xu Y. A Low fT3 Level as a Prognostic Marker in Patients With Acute Myocardial Infarctions. Internal Med (2012) 51(21):3009–15. 10.2169/internalmedicine.51.7902 23124142

[26] Abdu FA Liu L Mohammed AQ Luo Y Xu S Auckle R. Myocardial Infarction With non-Obstructive Coronary Arteries (MINOCA) in Chinese Patients: Clinical Features, Treatment and 1year Follow-Up. Int J Cardiol (2019) 287:27–31. 10.1016/j.ijcard.2019.02.036 30826195

[27] Lindahl B Baron T Erlinge D Hadziosmanovic N Nordenskjold A Gard A. Medical Therapy for Secondary Prevention and Long-Term Outcome in Patients With Myocardial Infarction With Nonobstructive Coronary Artery Disease. Circulation (2017) 135(16):1481–9. 10.1161/CIRCULATIONAHA.116.026336 28179398

[28] Wang B Liu S Li L Yao Q Song R Shao X. Non-Thyroidal Illness Syndrome in Patients With Cardiovascular Diseases: A Systematic Review and Meta-Analysis. Int J Cardiol (2017) 226:1–10. 10.1016/j.ijcard.2016.10.039 27776249

[29] Yuan D Zhang C Jia S Liu Y Jiang L Xu L. Predictive Value of Free Triiodothyronine (FT3) to Free Thyroxine (FT4) Ratio in Long-Term Outcomes of Euthyroid Patients With Three-Vessel Coronary Artery Disease. Nutr Metab Cardiovasc Dis (2021) 31(2):579–86. 10.1016/j.numecd.2020.10.011 33250369

[30] Yamazaki Y Shoji T Miyashima M Nagata Y Kakutani Y Ochi A. Low Free Triiodothyronine Level as a Predictor of Cardiovascular Events and All-Cause Mortality in Patients Undergoing Hemodialysis: The DREAM Cohort. J Atheroscler Throm (2020). 10.5551/jat.60624 PMC8560844 33361647

[31] Mitchell J Hellkamp A Mark D Anderson J Johnson G Poole J. Thyroid Function in Heart Failure and Impact on Mortality. JACC Heart failure (2013) 1(1):48–55. 10.1016/j.jchf.2012.10.004 24159562 PMC3803999

[32] Okayama D Minami Y Kataoka S Shiga T Hagiwara N. Thyroid Function on Admission and Outcome in Patients Hospitalized for Acute Decompensated Heart Failure. J Cardiol (2015) 66(3):205–11. 10.1016/j.jjcc.2015.04.006 25982671

[33] Bunevicius A Deltuva V Tamasauskas S Tamasauskas A Laws ER Jr. Bunevicius R. Low Triiodothyronine Syndrome as a Predictor of Poor Outcomes in Patients Undergoing Brain Tumor Surgery: A Pilot Study: Clinical Article. J Neurosurg (2013) 118(6):1279–87. 10.3171/2013.1.jns121696 23480214

[34] Scoscia E Baglioni S Eslami A Iervasi G Monti S Todisco T. Low Triiodothyronine (T3) State: A Predictor of Outcome in Respiratory Failure? Results of a Clinical Pilot Study. Eur J Endocrinol (2004) 151(5):557–60. 10.1530/eje.0.1510557 15538932

[35] Kong S Kim J Park Y Lee J Hong A Shin C. Low Free T3 to Free T4 Ratio was Associated With Low Muscle Mass and Impaired Physical Performance in Community-Dwelling Aged Population. Osteoporos Int (2020) 31(3):525–31. 10.1007/s00198-019-05137-w 31784788

[36] Anderton TS Saunderson CED Jain M Sengupta A Bernstein BS Halliday B. A Case Report of Acute Myopericarditis Associated With Graves’ Thyrotoxicosis. Eur Heart J - Case Rep (2020) 4(6):1–5. 10.1093/ehjcr/ytaa465 PMC7793046 33442602

[37] Krishnan GD Yahaya N Yahaya M. Hyperthyroidism Presenting as ST Elevation Myocardial Infarction With Normal Coronaries – A Case Report. J ASEAN Fed Endocrine Societies (2019) 34(1):92–4. 10.15605/jafes.034.01.14 PMC7784089 33442142

