# Supplementary material for: Low Free Triiodothyronine as a Predictor of Poor Prognosis in Patients With Myocardial Infarction With Non-Obstructive Coronary Arteries
Source: Front Endocrinol (Lausanne). 2021 May 31;12:681978. doi: 10.3389/fendo.2021.681978 (PMC8202072; doi:10.3389/fendo.2021.681978)
Supplement: Supplementary file 1 [file Table_1.docx]

Supplemental table 1**|** Subgroup analysis of the association between fT3 levels and MACE.

| Factors | Subgroup | HR (95% CI) | Interaction P-value |
| --- | --- | --- | --- |
| Age | **<**65 years  ≥65 years | 0.55 (0.25 - 1.22)  0.86 (0.47 - 1.58) | 0.390 |
| Sex | Male  Female | 0.46 (0.22 - 0.93)  0.95 (0.57 - 1.59) | 0.470 |
| Hypertension | Yes  No | 0.99 (0.44 - 2.24)  0.52 (0.26 - 1.04) | 0.228 |
| Diabetes mellitus | Yes  No | 0.21 (0.03 - 1.51)  0.77 (0.47 - 1.26) | 0.259 |
| BMI | ≥23.1 kg/m2  <23.1 kg/m2 | 0.94 (0.60 - 1.45)  0.52 (0.24 - 1.11) | 0.177 |
| Smoking | Yes  No | 0.72 (0.36 - 1.44)  0.66 (0.33 - 1.33) | 0.879 |
| Atrial fibrillation | Yes  No | 0.71 (0.08 - 1.48)  0.69 (0.42 - 1.15) | 0.984 |
| LVEF | **<**50  ≥50 | 0.63 (0.27 - 1.43)  0.74 (0.36 - 1.49) | 0.672 |
| cTnT | low  high | 0.86 (0.54 - 1.37)  0.26 (0.08 - 0.85) | 0.096 |
| NT-proBNP | ≥400 pg/ ml  <400 pg/ml | 0.76 (0.39 - 1.43)  0.73 (0.32 - 1.71) | 0.981 |
| Vessel with any stenosis | 1-2vessel disease  3 vessel disease | 0.94 (0.61 - 1.45)  1.66 (0.33 - 8.39) | 0.514 |

BMI, body mass index; LVEF, left ventricular ejection fraction; cTnT, cardiac troponin T; NT-proBNP, N-terminal pro-brain natriuretic peptide; HR, hazard ratio; CI, confidence interval.
